# Barium Sulfate Deposition in the Gastrointestinal Tract: Review of the literature

**DOI:** 10.1186/s13000-022-01283-8

**Published:** 2022-12-31

**Authors:** Daniel J. Zaccarini, David Lubin, Soma Sanyal, Jerrold L. Abraham

**Affiliations:** 1 Departments of Pathology, State University of New York (SUNY), Upstate Medical University, Syracuse, NY USA; 2 Departments of Radiology, State University of New York (SUNY), Upstate Medical University, Syracuse, NY USA

**Keywords:** Barium sulfate, Granular and rhomboid deposition

## Abstract

**Background:**

Barium sulfate is utilized for imaging of the gastrointestinal tract and is usually not deposited within the wall of the intestine. It is thought that mucosal injury may allow barium sulfate to traverse the mucosa, and allow deposition to occur uncommonly. Most pathology textbooks describe the typical barium sulfate deposition pattern as small granular accumulation in macrophages, and do not describe the presence of larger rhomboid crystals. This review will summarize the clinical background, radiographic, gross, and microscopic features of barium sulfate deposition in the gastrointestinal tract. A review of the PubMed database was performed to identify all published cases of barium sulfate deposition in the gastrointestinal tract that have been confirmed by pathologic examination.

**Conclusions:**

A review of the literature shows that the most common barium sulfate deposition pattern in the gastrointestinal tract is finely granular deposition (30 previously described cases), and less commonly large rhomboid crystals are seen (19 cases) with or without finely granular deposition. The fine granules are typically located in macrophages, while rhomboid crystals are usually extracellular. There are various methods to support that the foreign material is indeed barium sulfate, however, only a minority of studies perform ancillary testing. Scanning electron microscopy with energy dispersive X-ray spectroscopy (SEM/EDS) can be useful for definitive confirmation. This review emphasizes the importance of recognizing both patterns of barium sulfate deposition, and the histologic differential diagnosis.

**Supplementary Information:**

The online version contains supplementary material available at 10.1186/s13000-022-01283-8.

## Clinical Background

Barium sulfate is commonly used to radiographically examine the intestines, and improve visualization by opacifying areas of interest fluoroscopically. Typically, this is done by either infusing a water-soluble contrast or using barium. Barium sulfate can be given orally or rectally, and was initially commonly used in the beginning of the twentieth century; substituting for a prior mixture of gruel and bismuth called “Rieder meal”[1]. One indication for single contrast enema is if the patient is unable to change positions on the exam table for a double contrast study [2]. Additionally, single contrast enema is used if only the position and length of a suspected stricture is needed to be evaluated, for lesions greater than 1 cm in size, to evaluate acute diverticulitis, or to evaluate for a colonic fistula [2]. Double contrast refers to the concomitant use of a negative contrast agent such as air or CO2 and a positive contrast agent such as barium. A double contrast enema is chosen especially if evaluation of the mucosa is desired.

For the evaluation and diagnosis of suspected tracheoesophageal fistulas, fluoroscopic esophagram with dilute barium contrast is used. Water soluble contrast with iodine should be avoided due to the risk of pulmonary edema and pneumonitis from hypertonic iodinated contrast [3, 4]. Fatal aspiration of barium can occur, albeit rarely [5]. After resolution of acute diverticulitis, patients may be administered barium to assess the extent of diverticula, and also to rule out other conditions that may mimic the clinical presentation [6].

Barium, by itself, is very adsorbent, and therefore is coated with agents such as methyl cellulose to help it remain in suspension [1]. Current preparations contain combinations of polysorbate 80, saccharin sodium, sodium benzoate, and benzoic acid [11]. Barium deposition in the colon was first described as a barium granuloma in 1954 by Beddoe et al. [12]. Barium granuloma is also known as *barytoma* or *barioma* [13]. Most of the time Barium sulfate is excreted in the feces without complication. It is generally believed that barium sulfate traverses the colonic mucosa when there is preexisting mucosal damage [14].

The risks associated with barium enema include colonic perforation, fecal impaction, and constipation. After perforation, barium, bacteria, and admixed feces in the peritoneum may lead to a severe peritonitis with high risk of mortality in up to half of patients [7]. Peritoneal barium can cause a severe inflammatory reaction, and treatment can involve lavage with a large volume of normal saline. Barium can remain and form deposits following a case of peritonitis, and can persist sometimes for years leading to fibrosis [8]. Additionally, in patients with other risk factors for small bowel obstruction, barium sulfate may contribute to additional risk for obstruction [9]. Moreover, there are rare reports of appendicitis after barium enema, so called “barium-induced appendicitis”; possibly from retention leading to obstruction of the appendiceal lumen [6, 15]. One case report described intravenous embolization of barium leading to fatality [10]. Therefore, after a pathologic diagnosis of barium sulfate deposition is rendered, it may provide etiologic clues to the occurrence of prior clinical events such as bowel perforation or obstruction.

### Radiographic findings

**Fig. 1:**
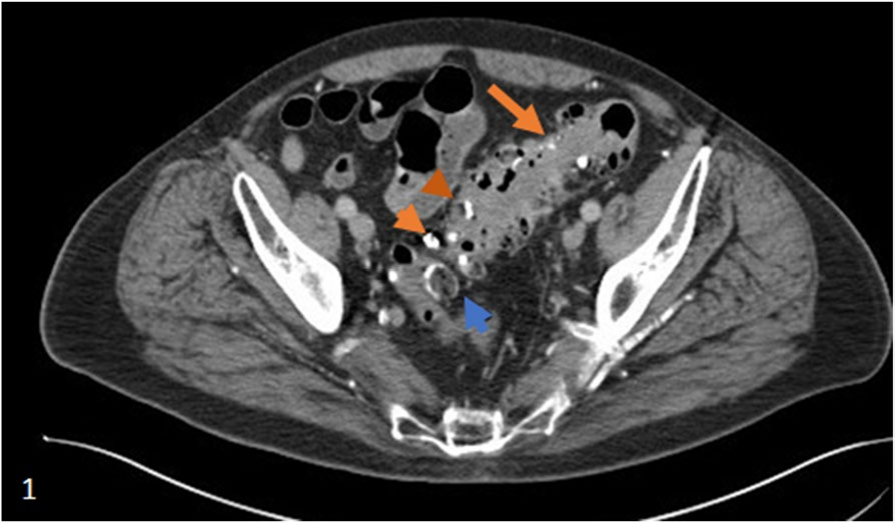
Axial CT image of the pelvis showing multiple sigmoid colonic diverticula (orange arrow). Many of these diverticula demonstrate markedly hyperattenuating material filling them (orange arrowheads) which was proven to be barium sulfate after resection of the colon. There is a sigmoid anastomotic donut in trans-axial section (blue arrow) with hyperattenuating material within it as well. Evidence of classic gross barium material along the peritoneal surfaces was not seen in this case

Barium deposition within the muscular wall of the colon can produce a unique transverse striated appearance on radiography owing to the unique anatomy of the inner circular layer of smooth muscle [13]. If the collection is subserosal in location, a lucent band may be seen [13]. Example of a case of barium deposition in the colon on axial CT image (Fig. 1). A number of findings are seen with bowel perforation following a barium enema. Barium is highly radiopaque and barium peritonitis can demonstrate radiopaque contrast coating the surfaces of the liver, bowel, and subdiaphragmatic surfaces.

### Gross pathology

On gross or endoscopic examination, barium granulomas may manifest as firm macules, plaques, or nodules that have a “scab-like”, ulcerated, or smooth appearance [16]. Gross lesions can be clinically worrisome for malignancy. Some lesions that are present deeper in the intestinal wall may not be readily apparent from the mucosal surface. A literature review (Additional file 1: Table S1) of 49 cases with pathologic confirmation shows that most cases in the gastrointestinal tract occur in the rectum (32), followed by rectosigmoid (3), sigmoid (3), transverse colon (3), stomach (2), and one case each in the appendix, esophagus, descending colon, jejunum, and colon (not otherwise specified). Lesions grossly range from 0.3 cm to 10 cm, and average 2.3 cm (Additional file 1: Table S1).

### Microscopic features

**Fig. 2:**
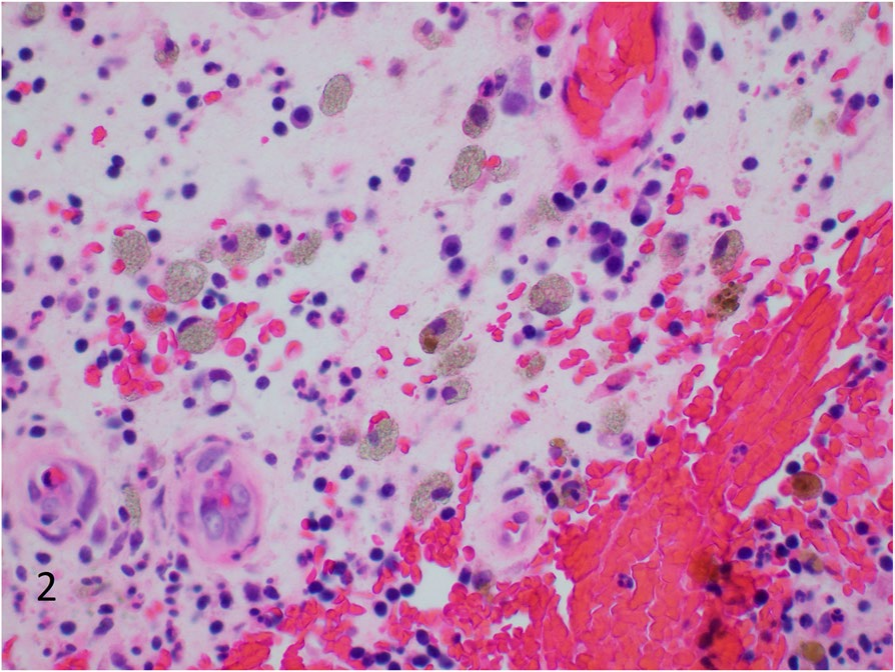
Histiocytes containing finely granular grey-brown material that was later confirmed to be barium sulfate. Background of granulation tissue with acute and chronic inflammation (H&E, 400 × magnification, 298 micron field width)

**Fig. 3:**
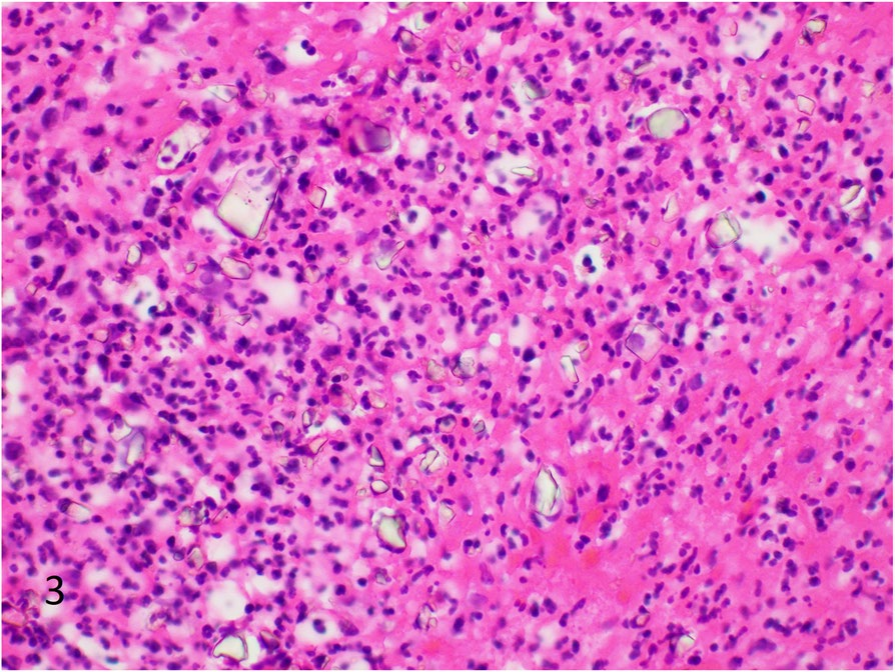
Rhomboid crystals of barium sulfate in a background of acute inflammation (H&E, 400 × magnification, 298 micron field width)

There are numerous brands of barium sulfate available in the market for clinical use. Levison et al. in 1984 analyzed many different types of barium sulfate used in two local hospitals. These included Micropaque® (Nicholas Aspro), E-Z-HD® (E-Z-EM Co Inc, Westbury, New York), Unibaryt® (Rotim Pharma, GmbH Weiterstadt, West Germany), Polibar ACB®, and Baritop 100® (Concept Pharmaceuticals Ltd). Most of the brands demonstrated small particles with weak birefringence. They found a less recognized deposition pattern with E-Z-HD, in the form of large rhomboid crystals [1]. A number of other studies have also described rhomboid crystals, and Additional file 1: Table S1 shows a review of the English literature on barium sulfate deposition (including only cases with pathologic examination) [13, 17–39]. Of the 49 prior cases, 30 cases described finely granular deposition, and 19 cases described deposition of larger rhomboid crystals with or without concurrent finely granular deposition. This illustrates that the most common pattern of deposition is a finely granular form. Most pathology textbooks describe a granular deposition pattern, and do not mention rhomboid crystals [40, 41]. If the reaction is in its early phase, there is acute inflammation and granulation tissue surrounding barium [1]. Examples of finely granular deposition in macrophages (Fig. 2), and rhomboid crystals (Fig. 3) in cases of barium sulfate deposition.

### Ancillary testing

**Fig. 4:**
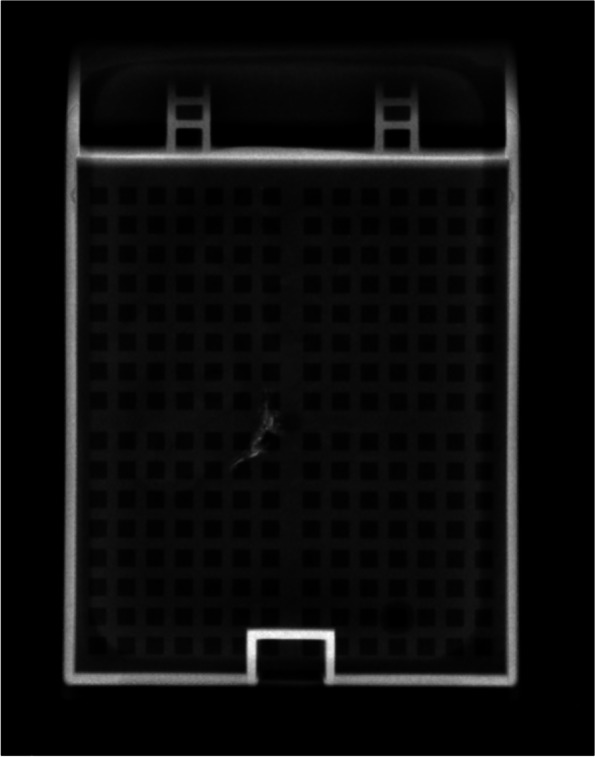
Radiograph of paraffin block showing opaque material in a case of barium sulfate deposition in the colon

**Fig. 5:**
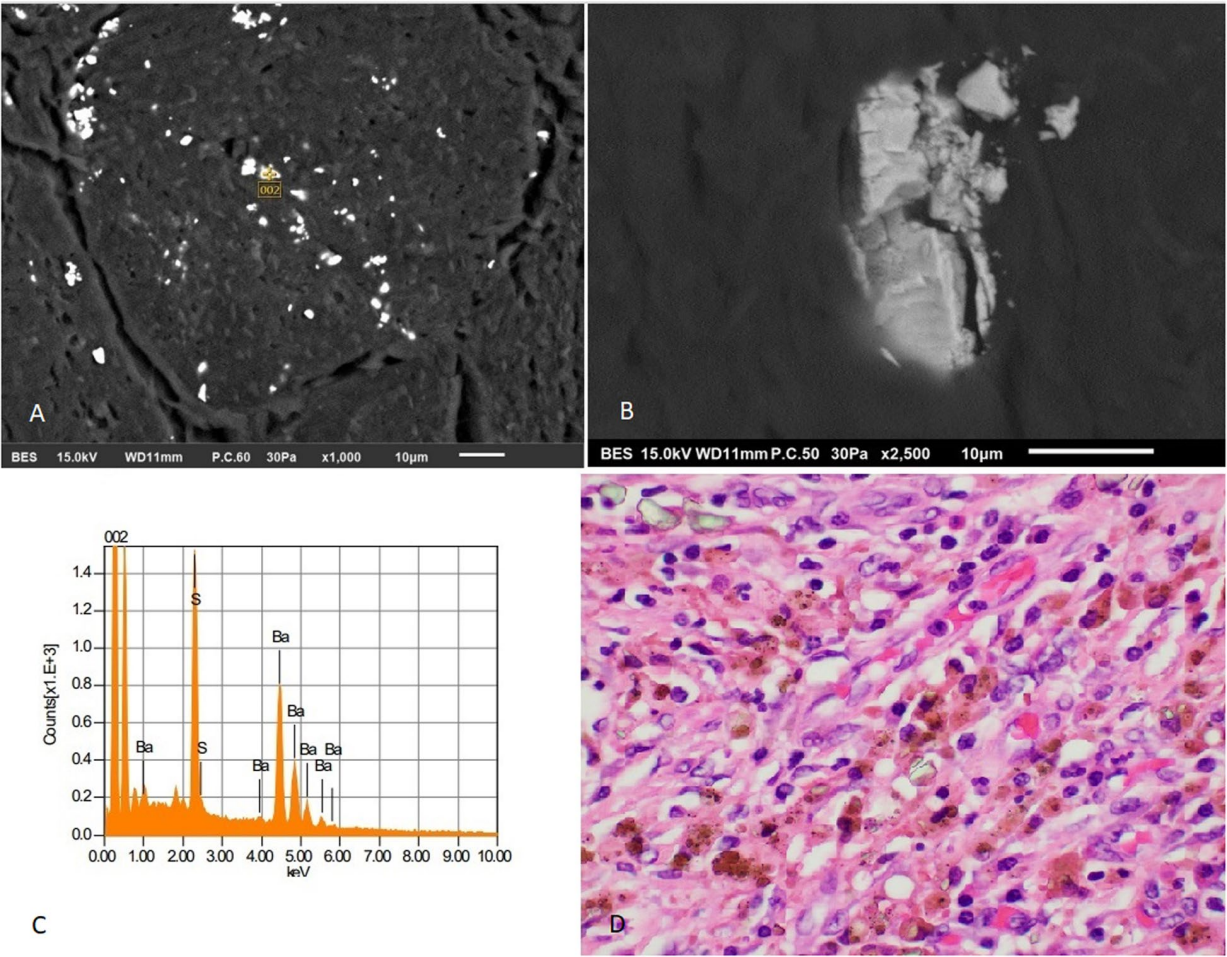
Scanning electron microscopy identifies the foreign material using backscattered electron imaging at 1000x (A) and detail of a fragmenting particle at 2500x (B). Note that the large particles may break down to tiny submicrometer particles often seen scattered in histiocytes by light microscopy. The chemical identification of the particles as Barium sulfate is confirmed by energy dispersive x-ray spectroscopy (EDS) (C), showing peaks for Barium and sulfur. High power view of rhomboid crystals in this case of barium sulfate deposition (D) (H&E, 400 × magnification, 298 micron field width)

It may be difficult to confirm if a foreign material is indeed barium sulfate by only using light microscopy. Various methodologies for confirmation have been described in the literature. Staining for rhodizonate can help support that the material may represent barium sulfate by looking for the presence of brown–red precipitate; however, other material such as lead, mercury, and strontium may also produce positivity [1]. Nonetheless, this stain is not commonly available. Radiography of the paraffin block will show that the material is opaque on x-ray (Fig. 4) [42]. Radiography of paraffin blocks is more widely available, and is a helpful tool when there is an appropriate clinical history. Scanning electron microscopy with energy dispersive X-ray spectroscopy (SEM/EDS) can be very useful for confirmation since it is highly specific; however, it is not routinely available in general pathology laboratories (Fig. 5).

### Differential diagnosis

**Fig. 6:**
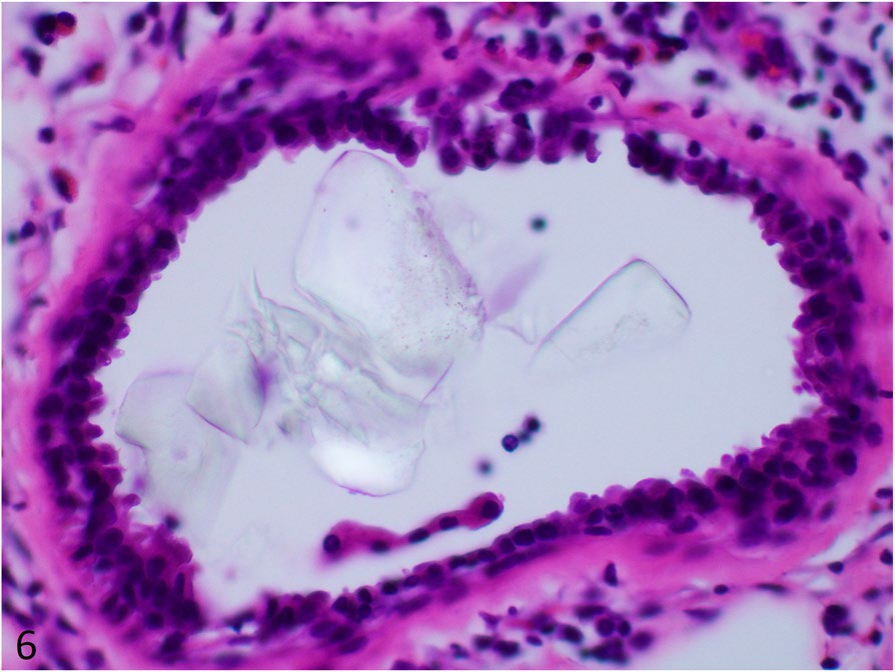
Irregularly shaped calcium oxalate crystals from a breast excision (H&E, 400 × magnification, 298 micron field width)

**Fig. 7:**
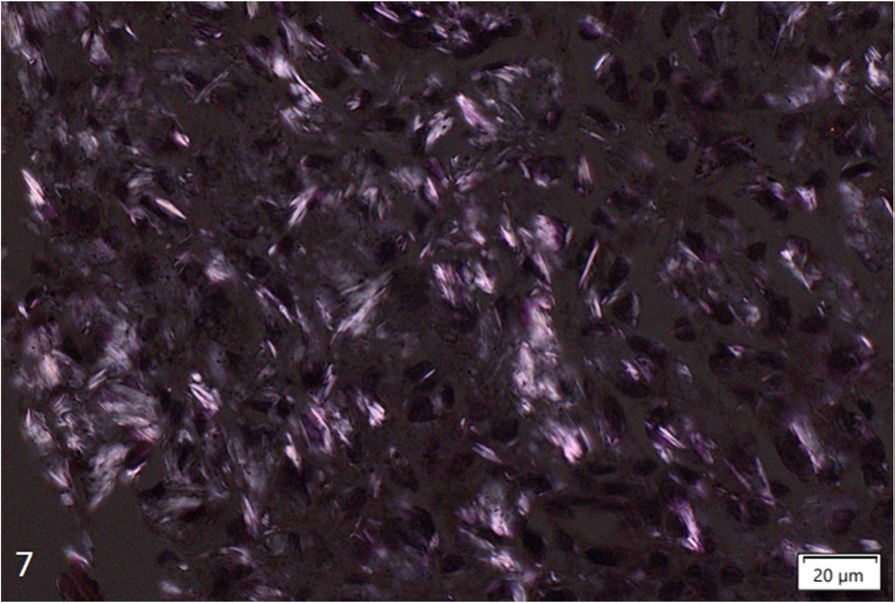
Talc particles with plate-like appearance from a transbronchial biopsy related to inhalation (H&E)

**Fig. 8:**
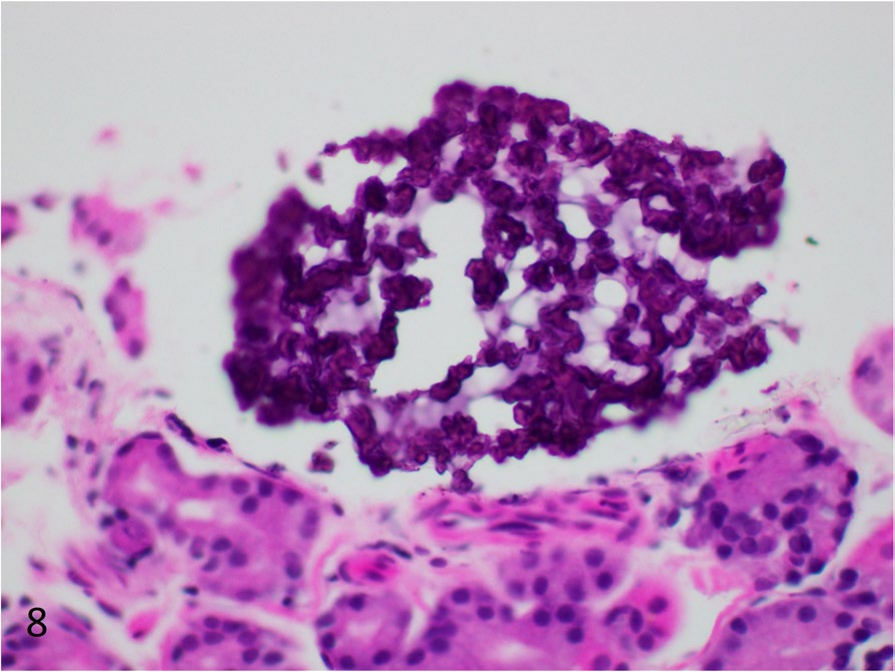
Crospovidone deposition showing darker purple on the outside and lighter purple on the inner aspects of the material (H&E, 400 × magnification, 298 micron field width)

**Fig. 9:**
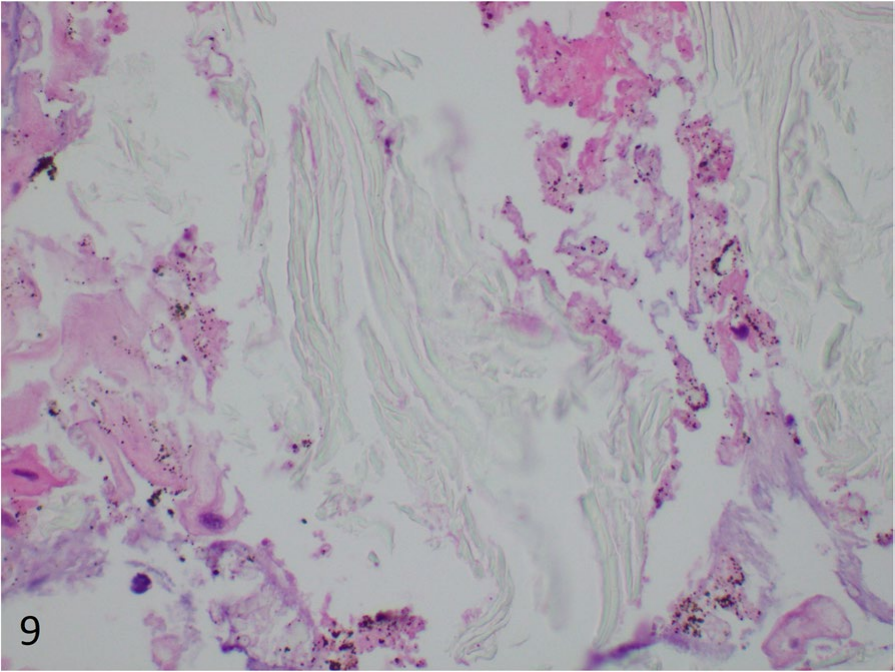
Microcrystalline cellulose with an elongated flakey appearance (H&E, 400 × magnification, 298 micron field width)

The morphologic differential diagnosis for large barium crystals includes calcium oxalate, calcium phosphate, talc, and medication fillers. Calcium phosphate has a purple appearance on routine histology which helps differentiate it from large barium crystals. Calcium oxalate crystals are irregularly shaped, birefringent, and admittedly difficult to discern from barium sulfate (Fig. 6). The presence of finely pigmented grey-brown macrophages are not expected with calcium oxalate, albeit may not always be present. Talc is platy and needle-like in appearance with polarization (Fig. 7). Medication fillers such as crospovidone have a coral shaped two-toned purple appearance in the gastrointestinal tract, and is not birefringent (Fig. 8) [43]. In contract, microcrystalline cellulose is rod-like or has a flake appearance, is clear, birefringent, and positive with GMS stain (Fig. 9) [43].

The finely granular form of barium sulfate deposition has a unique grey-brown appearance. Melanosis coli and iron-laden macrophages may enter the differential in this scenario, however, they do not show the unique grey appearance. An area of concern for the future is the increasing application of digitally scanned slides for routine diagnosis that will preclude the use of polarized light microscopy to detect birefringent material, and also limit the ability to resolve fine details.

## Conclusions

We describe commonly reported features of barium sulfate deposition in the gastrointestinal tract. The most common site of deposition is the rectum, and lesions average 2.3 cm in size. Barium granulomas often contain histiocytes with finely pigmented material, though large rhomboid crystals are also an important feature to recognize. A clinical history of barium radiography may be helpful to clue into the diagnosis, however confirmation with SEM/EDS is helpful for definitive diagnosis.

## Supplementary Information

**Additional file 1. Table S1** A literature review of 49 cases of barium sulfate deposition in the gastrointestinal tract with pathologic confirmation.

## Data Availability

All data generated or analyzed during this study are included in this published article.

## Abbreviations

CT: Computed Tomography
SEM/EDS: Scanning Electron Microscopy with Energy Dispersive X-ray Spectroscopy
