# Supplementary figures and images for: Barium Sulfate Deposition in the Gastrointestinal Tract: Review of the literature
Source: Diagn Pathol. 2022 Dec 31;17:99. doi: 10.1186/s13000-022-01283-8 (PMC9805050; doi:10.1186/s13000-022-01283-8)

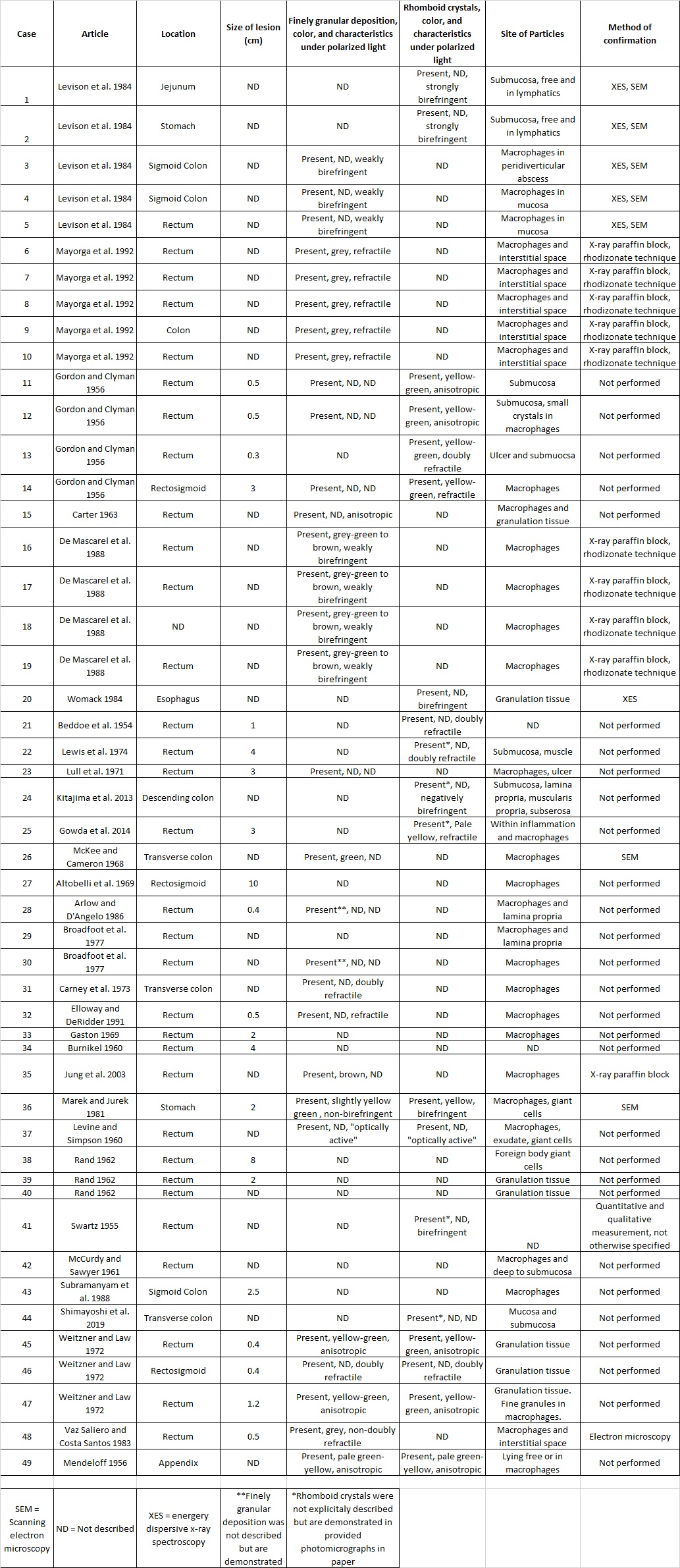

Supplement: Supplementary file 1 — Additional file 1. Table S1 A literature review of 49 cases of barium sulfate deposition in the gastrointestinal tract with pathologic confirmation. [file 13000_2022_1283_MOESM1_ESM.tif]
